# *TYR* Gene in Llamas: Polymorphisms and Expression Study in Different Color Phenotypes

**DOI:** 10.3389/fgene.2019.00568

**Published:** 2019-06-12

**Authors:** Melina Anello, Estefanía Fernández, María Silvana Daverio, Lidia Vidal-Rioja, Florencia Di Rocco

**Affiliations:** ^1^Laboratorio de Genética Molecular, Instituto Multidisciplinario de Biología Celular, CONICET–UNLP–CIC, La Plata, Argentina; ^2^Cátedra de Biología, Departamento de Ciencias Biológicas, Facultad de Ciencias Exactas, Universidad Nacional de La Plata, La Plata, Argentina

**Keywords:** tyrosinase, coat color, dilution, *Lama glama*, polymorphisms, expression

## Abstract

Tyrosinase, encoded by *TYR* gene, is an enzyme that plays a major role in mammalian pigmentation. It catalyzes the oxidation of L-dihydroxy-phenylalanine (DOPA) to DOPA quinone, a precursor of both types of melanin: eumelanin and pheomelanin. *TYR* is commonly known as the *albino* locus since mutations in this gene result in albinism in several species. However, many other *TYR* mutations have been found to cause diluted phenotypes, like the Himalayan or chinchilla phenotypes in mice. The llama (*Lama glama*) presents a wide variety of coat colors ranging from non-diluted phenotypes (eumelanic and pheomelanic), through different degrees of dilution, to white. To investigate the possible contribution of *TYR* gene to coat color variation in llamas, we sequenced *TYR* exons and their flanking regions and genotyped animals with diluted, non-diluted, and white coat, including three blue-eyed white individuals. Moreover, we analyzed mRNA expression levels in skin biopsies by qPCR. *TYR* coding region presented nine SNPs, of which three were non-synonymous, c.428A > G, c.859G > T, and c.1490G > T. We also identified seven polymorphisms in non-coding regions, including two microsatellites, an homopolymeric repeat, and five SNPs: one in the promoter region (c.1-26C > T), two in the 3′-UTR, and two flanking the exons. Although no complete association was found between coat color and SNPs, c.1-26C > T was partially associated to diluted phenotypes. Additionally, the frequency of the G allele from c.428A > G was significantly higher in white compared to non-diluted. Results from qPCR showed that expression levels of TYR in white llamas were significantly lower (*p* < 0.05) than those in diluted and non-diluted phenotypes. Screening for variation in regulatory regions of TYR did not reveal polymorphisms that explain such differences. However, data from this study showed that TYR expression levels play a role in llama pigmentation.

## Introduction

In mammals, basic coat colors are defined by the relative proportion between two types of melanin: eumelanin (black or brown) and pheomelanin (yellow or red). At molecular level, eumelanin:pheomelanin ratio is regulated mainly by the ligand–receptor system of the agouti signaling protein (*ASIP*) and the melanocortin 1-receptor (*MC1R*). Additionally, color phenotype depends on the expression and interaction of many other genes that can disrupt the normal pigmentation pathway (Cieslak et al., 2011).

Tyrosinase, encoded by *TYR* gene, is a key enzyme for melanin synthesis. This copper-containing enzyme catalyzes the first two steps in the melanin biosynthesis pathway, converting tyrosine to L-dihydroxy-phenylalanine (DOPA) and afterward DOPA to DOPA quinone, a precursor of both types of melanin. *TYR* is commonly known as the albino locus since mutations in this gene result in albinism in several species, including humans. More than 100 mutations in the *TYR* gene have been identified in people with oculocutaneous albinism type 1 (The Albinism Database^1^). Because of the lack of melanin production, these patients present white hair, light-colored eyes, and a very pale skin that does not tan. Albinism has also been described in mice (Yokoyama et al., 1990; Beermann et al., 2004), cats (Imes et al., 2006), cattle (Schmutz et al., 2004), rabbits (Aigner et al., 2000), buffalos (Damé et al., 2012), donkeys (Utzeri et al., 2016) among other species.

Additionally, other *TYR* mutations have been found to cause milder phenotypes. For example, the Himalayan phenotype implicates different mutations that result in a temperature-regulated activity of *TYR*, where cooler parts of the body are pigmented while warmer parts remain white. These mutations have been described in mice (Kwon et al., 1989), minks (Benkel et al., 2009), rabbits (Aigner et al., 2000), and cats (Lyons et al., 2005). Another example is the chinchilla allele from mice, which encodes a tyrosinase whose activity is from one-third to one-half that of the normal. This is caused by a point mutation in the *TYR* gene and chinchilla mice exhibit a grayish color (Lamoreux et al., 2001). Another point mutation in *TYR* gene is responsible for the mice platinum phenotype, which results in animals with almost complete loss of pigmentation (Orlow et al., 1993). Recently, two near-white *TYR* alleles were described in mice, Dhoosara and Chandana, characterized by a marked hypopigmentation in the body and the eyes (Challa et al., 2016).

Most of what is known about the regulation of *TYR* gene derives from studies carried out in mice and extended to human (Giraldo et al., 2003; Murisier and Beermann, 2006; Reinisalo et al., 2012). According to these studies, *TYR* is regulated by a combination of proximal promoter elements and far upstream regulatory sequences, being the locus control region (LCR) the most important one.

The llama (*Lama glama*) is a South American camelid which presents a great diversity of coat colors, including black, chocolate brown, many shades of light brown (from red to pale cream), and complete white coat (Frank et al., 2006). Occasionally, some white llamas present blue eyes. Patterns and spotted phenotypes are also frequent in llamas.

The molecular mechanisms that control pigmentation in camelids have mainly been studied in alpacas (*Vicugna pacos*). Different authors have studied *MC1R* (Powell et al., 2008; Feeley and Munyard, 2009; Guridi et al., 2011; Chandramohan et al., 2015), *ASIP* (Feeley et al., 2011; Chandramohan et al., 2013), *TYR*, and *MATP* genes (Cransberg and Munyard, 2011) in relation to alpaca coat colors. However, in llamas the mechanisms that control pigmentation are not fully understood. In a previous work, we analyzed *MC1R* and *ASIP* genes and found two *ASIP* polymorphisms associated with eumelanic phenotype (Daverio et al., 2016). We also studied *KIT* and *MITF-M* genes and their relation to white coat. Although no variants were found to be associated with white phenotype, both genes were less expressed in this phenotype (Anello et al., 2019). Here, we aimed to study the possible contribution of *TYR* gene to coat color variation in llamas and to do so, we describe the *TYR* gene, its variation, and its skin expression.

## Materials and Methods

### Samples

All samples were collected following the recommendations of the Argentine Ethical Guidelines for Biomedical Investigation in Animals from Laboratory, Farm, or Obtained from Nature (Resolution No. 1047/05 from CONICET, Argentina). The protocol was approved by the Institutional Committee for the Care and Use of Laboratory Animals (CICUAL) from the Multidisciplinary Institute of Cellular Biology (IMBICE). All llama owners provided an informed consent for their animal’s inclusion prior to sampling and were present during the collection of the samples.

To determine *TYR* sequence and variation in llamas, blood samples were collected from 29 unrelated animals from different breeding farms in Argentina. Eighty-five additional samples were included for genotyping of polymorphisms of interest. All samples were taken by jugular vein puncture from young-adult animals. Approximately equal sex proportions were sampled and the lack of relationship between the sampled animals was verified by consulting breeder records. Coat color phenotype for each sample was documented and pictures were taken whenever possible.

**FIGURE 1:**
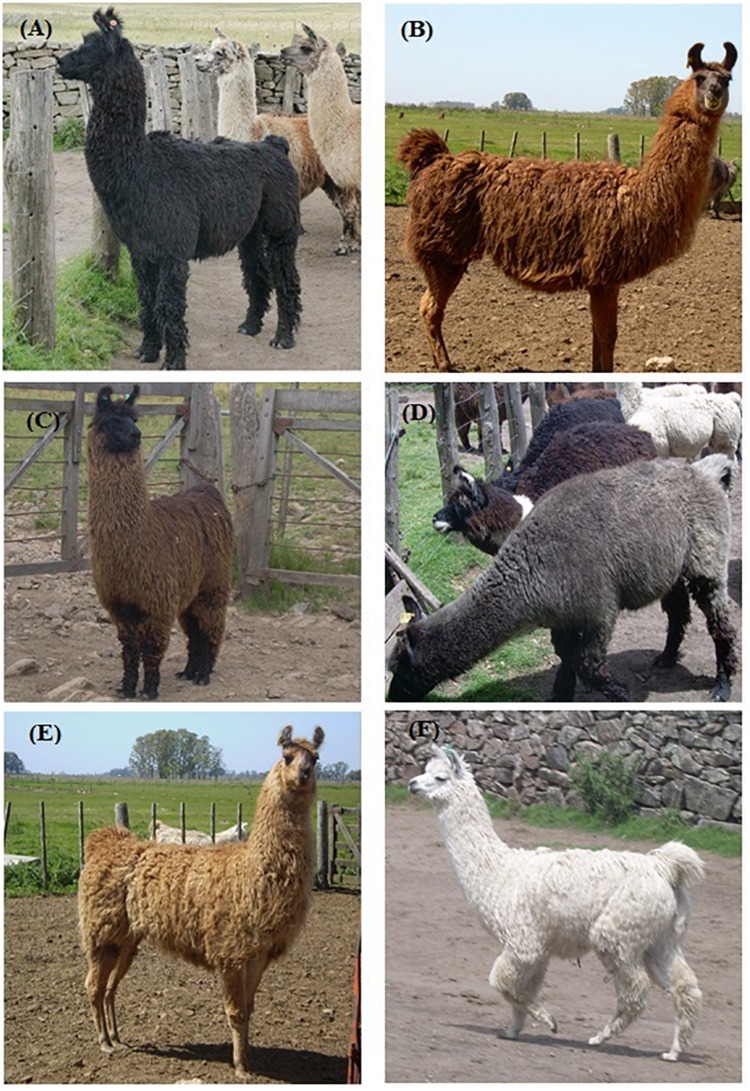
Photographs of llamas illustrating color phenotypes included in this study. **(A)** Black, **(B)** Red, **(C)** Black Face, **(D)** Gray, **(E)** Fawn, and **(F)** White.

Phenotypes sampled were divided into three groups for the subsequent analyses: NON-DILUTED (*n* = 47), DILUTED (*n* = 27), and WHITE (*n* = 40). The first group included the following phenotypes: BLACK, eumelanic llamas with non-diluted black or dark brown pigmented coats; RED, llamas with pheomelanic red coat, completely pigmented; and BLACK FACE, animals that presented non-diluted pheomelanic pigmentation with black face and legs, a very common phenotype among llamas. On the contrary, DILUTED PHENOTYPES showed either a eumelanin or pheomelanin dilution, so it included GRAY, individuals with diluted eumelanic coats, and FAWN, llamas with diluted pheomelanin (light brown, fawn, and cream coats). Finally, WHITE included two phenotypes, WHITE, non-albino llamas with dark eyes and a full white coat; and BLUE-EYED WHITE, individuals with full white coat and blue eyes. Figure 1 shows some examples of llama phenotypes included in this study. Pictures of the blue-eyed white phenotype are not available. More information about samples used in this paper can be found in Supplementary Table 1.

For the expression analysis of *TYR*, skin biopsies from pheomelanic NON-DILUTED, pheomelanic DILUTED, and WHITE llamas (three animals of each phenotype) were collected using a disposable 3-mm diameter biopsy punch. To avoid RNA degradation, samples were kept in RNAlater (SIGMA, Germany) during their transportation to the laboratory, where they were immediately processed.

### DNA Extraction and PCR Amplification

Total genomic DNA was extracted from blood samples using standard procedures (Sambrook and Russell, 2001). DNA was resuspended in TE buffer and stored at −20°C for further analysis.

Primers flanking each of the five exons were designed over the alpaca genome available at Genome Browser (GCA_000164845.2). Supplementary Table 2 shows primers sequences and amplicon length.

Additionally, two regions of *TYR* promoter were amplified in 15 samples. A 792-bp fragment corresponding to *TYR* proximal protomer region and a 789-bp fragment where the LCR was located, at approximately at −9 kb. Since information about this latter region was restricted to mouse and human (AF364302.1 and AY180962.1) we aligned them with genomes scaffolds from other camelids available at GenBank (*Camelus dromedarius* NW_011591148.1, *Camelus bactrianus* NW_011544909.1, *Camelus ferus* NW_006211950.1, and *V. pacos* NW_005883058.1) in order to designed primers (Supplementary Table 2).

Polymerase chain reaction amplification reactions were performed with Taq DNA polymerase (PB-L, Pegasus) and 1× PCR buffer (PB-L, Pegasus) according to manufacturer’s instructions. The cycling profile consisted of an initial denaturation step at 94°C for 3 min, 30 cycles of 40 s at 94°C, 50 s at 58–65°C, 40 s at 72°C, and a 5-min final extension at 72°C. PCR products were checked on a 1% agarose gel stained with GelRed^TM^, purified by PEG precipitation, and sequenced by Macrogen Inc., Korea.

The sequences obtained were aligned and analyzed to identify polymorphisms using Geneious software (v.6.1.8, Biomatters). Elements in the regulatory regions were identified by alignment with mouse and human sequences.

Protein topology and domains were predicted using Constrained Consensus TOPology prediction server (CCTOP) (Dobson et al., 2015) and Conserved Domains Database (CDD) (Marchler-Bauer et al., 2017) software, respectively, and compared with UniProt reviewed entries (Bateman et al., 2017). TMHMM server^2^ was used for the prediction of transmembrane regions. Llama TYR protein was aligned to the homologous proteins of the following species: *V. pacos* (XP_006218431.1), *Ovis aries* (NP_001123499.1), *Capra hircus* (NP_001274491.1), *Homo sapiens* (AAB60319.1), *Mus musculus* (AAA40516.1), *Desmodus rotundus* (XP_024428480.1), *Canis lupus familiaris* (NP_001002941), *Delphinapterus leucas* (XP_022449451), and *Neovison vison* (AJO15925.1).

### Genotyping and Analysis of Polymorphisms of Interest

Single-nucleotide polymorphisms c.1-26C > T and c.1490G > T were genotyped by allele-specific PCR amplification. For each variant, a pair of primers was designed with the corresponding complementary nucleotide in the 3′-end of the forward primer (Supplementary Table 2). Additionally, a destabilizing mismatch was introduced within the 3′-end of the allele-specific primers to improve specificity. Each reaction was carried out in a separate tube and allele products, which presented different lengths, were further identified by 2% agarose gel electrophoresis (for 90 min at 90 V) stained with GelRed^TM^. Positive sequenced controls for each variant determination were used.

The SNPs c.428A > G and c.859G > T were genotyped by PCR–RFLP using sequenced samples as controls. PCR products from exon 1 were digested with the enzyme *Rsa1* that recognizes the c.428A > G mutation. If the amplicon presented allele A, the enzyme cut it into two fragments of 818 and 140 bp while if the amplicon presented the G allele, a new restriction site was created and digestion resulted in three fragments of 506, 312, and 140 bp. Digestion mix consisted of 7 μl of PCR product, 5 U of *Rsa1* (Thermo Fisher Scientific Inc.), and 1.5 μl of 10× Buffer Tango. Digestions were incubated at 37°C overnight and restriction fragments were analyzed by electrophoresis on a 2% agarose gel for 90 min at 90 V, stained with GelRed^TM^. PCR products from exon 2 were digested with the enzyme *BsmAI* to genotype the SNP c.859G > T. If the product had the allele T, *BsmAI* cut it into two fragments of 433 and 88 bp while if it presented the G allele, it remained undigested. Digestion mix consisted of 7 μl of PCR product, 5 U of *BsmAI* (New England BioLabs Inc.), and 1.5 μl of 10× Buffer 3. Digestions were incubated at 55°C overnight and restriction fragments were analyzed by 8% acrylamide gel electrophoresis (180 min at 160 V) stained with GelRed^TM^.

Association between allelic frequencies or genotypes and phenotypes was determined by Fisher’s exact test or Monte Carlo method, depending on the data set distribution, using PASW Statistics 1.8 (SPSS Inc., 2009). Association between haplotypes for the three non-synonymous SNPs was performed using the same methods, including samples whose haplotypes could be determined without ambiguities.

Web servers PolyPhen-2^3^, SIFT^4^, and PROVEAN Protein^5^ were used to predict the possible impact of amino acid substitutions on TYR protein. PolyPhen-2 uses a sequence- and structure-based approach and classifies the SNPs as benign, possibly damaging, or probably damaging using a position-specific independent count score which ranges from 0 to 1. SIFT predicts whether an amino acid substitution affects protein function based on sequence homology and the physical properties of amino acids and classifies substitutions in tolerant or intolerant based on scores (damaging if the score is =0.05 and tolerated if the score is >0.05). PROVEAN Protein applies an evolutionary conservation-based method and scores the SNP as neutral or deleterious (numbers equal or below −2.5 are considered deleterious).

### RNA Extraction and Expression Analysis

Total RNA was extracted from skin biopsies by homogenization in TRIzol^®^ according to manufacturer’s instructions. Reverse transcription was performed to obtain cDNA, using RevertAid Reverse Transcriptase (Thermo Fisher Scientific Inc.), and random primers (Biodynamics), following the manufacturer’s instructions.

Quantitative real-time PCR was carried out using a pair of primers specifically designed for this assay, where one primer annealed over exon–exon junction, avoiding genomic amplification (Table 1). Ribosomal *18S*, which has been previously used in melanogenesis expression studies (Saravanaperumal et al., 2014), was used for data normalization.

**TABLE 1:** Distribution of genotypes among phenotypic groups.

		**c.1-26C/T**			**c.428A > G**			**c.859G > T**			**c.1490G > T**		
		**C/C**	**C/T**	**T/T**	**A/A**	**A/G**	**G/G**	**G/G**	**G/T**	**T/T**	**G/G**	**G/T**	**T/T**
NON-DILUTED PHENOTYPES	BLACK	20	3	–	22	1	–	13	4	6	17	6	1
	RED	7	2	1	9	1	–	8	1	1	8	1	1
	BLACK FACE	9	2	2	12	1	–	13	–	–	5	7	1
	TOTAL	36	7	3	43	3	–	34	5	7	30	14	3
DILUTED PHENOTYPES	FAWN	11	7	1	19	1	–	13	1	3	9	8	3
	GRAY	2	3	–	6	1	–	5	–	–	3	4	–
	TOTAL	13	10	1	25	2	–	18	1	3	12	12	3
WHITE PHENOTYPES	WHITE	33	4	–	29	6	2	26	5	6	26	10	1
	BLUE-EYED WHITE	1	2	–	2	1	–	3	–	–	1	2	–
	TOTAL	34	6	0	31	7	2	29	5	6	27	12	1

Amplification reactions were carried out in a RotorGene Q (Qiagen) and consisted of 20 μl, including 4 HOT FIREPol^®^ EvaGreen^®^ qPCR Mix Plus (ROX) (Solis Biodyne), 0.5 mM of each primer, and 1 ng cDNA. The cycling parameters were: 15 min at 95°C, 40 cycles of 15 s at 95°C, 20 s at 60°C, 20 s at 72°C, and a final gradient from 95 to 72°C. qPCR reaction were optimized; PCR efficiencies calculated from the slope were within 90–110%, *r*^2^ over 98%. Each gene was amplified in three technical replicates for every sample and two NTC controls. Melting curve analysis was performed following amplification to verify the absence of non-specific amplification or primer dimer. Ct was determined by Rotor-Gene Q Pure Detection software version 2.3.1. Quantification of transcript abundance was carried out using the comparative threshold cycle (Ct) method by Livak and Schmittgen (2001), and ANOVA was used to assess if differences in expression were significant.

## Results

### Description of *TYR* Gene

We sequenced the *TYR* five exons with its flanking regions, including 58 bp from the 5′-untranslated region (UTR) and 85 bp from the 3′-UTR. The entire coding sequence comprised 1593 bp, divided into: 819 bp for exon 1, 218 bp for exon 2, 147 bp for exon 3, 182 bp for exon 4, and 227 bp for exon 5. Llama *TYR* gene sequence was deposited in GenBank under the accession number MK089778.

The protein encoded by the *TYR* gene is predicted to have 530 amino acids. It presents a signal peptide of 18 residues, an EGF-like domain (from amino acid 57 to 113) and a transmembrane region (from 474 to 496). The characteristic tyrosinase domain that binds two copper ions, via two sets of three histidine, expands from amino acid 170 to 403 (Supplementary Figure 1). Identity to orthologous proteins was as high as 99% with alpaca, 91% with sheep and goat, 88% with human, and 85% with mouse.

### *TYR* Polymorphisms and Coat Color Phenotype

**FIGURE 2:**
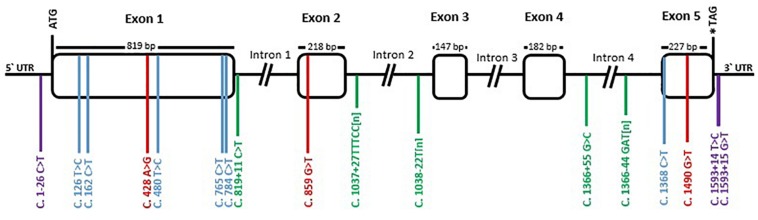
Schematic representation of *TYR* gene with its polymorphism’s distribution in llamas. Rectangular boxes correspond to exons while introns and non-translated regions are represented by black lines. Positions +1 was assigned to the translation initiation site (ATG codon) and ^*^ indicates the stop codon. Polymorphisms numbering is relative to the coding DNA sequence and indicated by transversal colored lines. Violet correspond to mutations in the UTR regions, blue correspond to synonymous mutation in the coding region, red to non-synonymous mutations, and green to intronic mutations.

To study the variation of *TYR* gene, the exons and their flanking regions were sequenced (3058 bp in total) in 29 llamas from different origins and with different color phenotypes. A total of 17 polymorphisms were detected. Figure 2 shows the distribution of polymorphisms along the gene. The coding region showed nine SNPs, three of which were non-synonymous: c.428A > G, located in exon 1, produces an amino acid change in the protein from histidine to arginine in residue 143 of the enzyme; c.859G > T was detected in exon 2 and it changes the alanine 287 to serine; and c.1490G > T, in exon 5, replaces the arginine in position 497 to leucine. Additionally, a C/T transition was observed 26 bp before the initial codon ATG and two consecutive SNPs were detected in the 3′-UTR region, 14 and 15 bp after the stop codon (c.1593+14T > C and c.1593+15G > T). Six synonymous SNPs were observed, in exons 1 and 5.

Furthermore, variation in the intronic regions was detected: two SNPs, two microsatellites, and one homopolymeric repeat were identified in the flanking regions of *TYR* exons (Figure 2). The first microsatellite was in intron 2, 27 bp after the end of exon 2, and consisted of five nucleotides (TTTCC) that repeated a variable number of times. Alleles variated in the number of repeats, between 20 and 30, and presented imperfections in their motif. According to the motif, we classified the alleles into three types: type 1 consisted of the five nucleotides TTTCC repeated, while type 2 added the pattern (TT)-(TTTCC)2-(TT) in between the repeats. Type 3 presented a 1 bp deletion at the beginning (TTTC) and a 1 bp substitution in some other repeats (TCTCC). This last allele was observed to segregate together with the T/T variant of SNP c.859G > T. An example of each type and a list of the observed alleles are provided in Supplementary Figure 2 and Supplementary Table 3. However, it is most likely that there exist more alleles, since there were heterozygous animals for which was not possible to determine the alleles by direct sequencing.

The second microsatellite was detected in intron 4, 44 bp before the start of exon 5 and it consisted of three nucleotides (GAT) that repeated 13–15 times. Occasionally, in some samples, one repeat of the motif GAT was replaced by AAT. The list presented in Supplementary Table 4 shows the observed genotypes and the possible combination of alleles.

Lastly, an homopolymeric repeat of thymidine was observed 22 bp before the start of exon 3. It presented variable length of 9 or 10 consecutive thymidine.

From all the variation found in *TYR* gene, we analyzed the polymorphisms that might influence coat color phenotype in 85 additional llama samples, that is the non-synonymous SNPs and the one located 26 bp upstream the ATG codon. Distribution of each genotype per color group is shown in Table 1. Regarding the coding SNPs, no significant association between any of the genotypes and color groups was found. However, distribution of allelic frequencies for the SNP c.428A > G was significantly different among groups (*p* = 0.015). The G allele was more frequent in the white group compared to non-diluted (*p* = 0.026) but the difference was not statistically different when compared to diluted (*p* = 0.103).

Furthermore, analysis of haplotypes for the three non-synonymous SNP did not show association with coat color (*p* > 0.05) (Supplementary Table 5).

Results from PolyPhen-2, SIFT, and PROVEAN Protein classified the three SNPs as benign/neutral/tolerated. The SNP c.428A > G deprives the protein of histidine 143, replacing it with another basic amino acid, arginine. Although this position is relatively conserved among species, bats also present an arginine residue (Supplementary Figure 1). The substitution c.859G > T encodes a change from alanine to serine in residue 287, a fairly common substitution (Supplementary Figure 1). Lastly, the SNP c.1490G > T changes arginine to leucine at amino acid 497, which is located at the end of the transmembrane region. This same mutation was reported in the alpaca *TYR* gene, and it was predicted to have a trivial biological effect (Cransberg and Munyard, 2011).

For the non-coding SNP c.1-26C/T, distribution among phenotypes resulted significantly different (*p* = 0.023). The heterozygous C/T genotype was significantly more frequent in the diluted group compared to non-diluted (*p* = 0.042) and white (0.013) (Table 1).

### Expression of Llama *TYR* in Different Color Phenotypes

**FIGURE 3:**
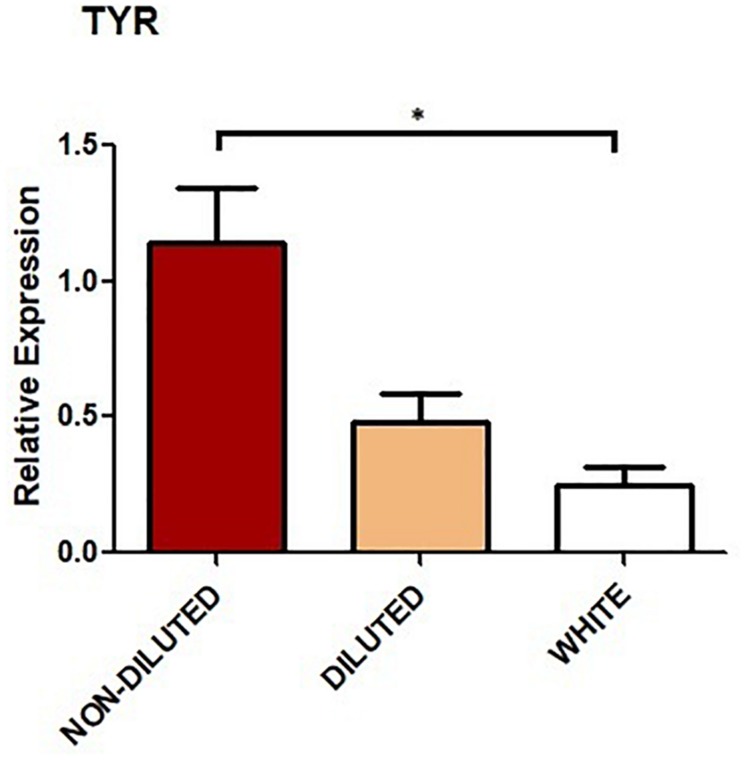
Relative expression of *TYR* in the skin of llamas. Bar graphs show mean values. Error bars represent standard error (*SE*). Three biological replicates were analyzed for each color group. Asterisk indicates statistically significant differences (*p* < 0.05).

Housekeeping gene *18S* and *TYR* amplicons were observed in every sample analyzed and melting curves confirmed a single product in each PCR. NTC controls showed no amplification. Expression levels of *TYR* for NON-DILUTED, DILUTED, and WHITE phenotypes were compared. Results showed that the expression level in the WHITE group was significantly lower (*p* < 0.05) than in the NON-DILUTED group. Although expression level was lower in WHITE llamas compared to DILUTED, this difference was not significant. Neither was the difference in expression between NON-DILUTED and DILUTED (Figure 3).

### Regulatory Regions of *TYR* Gene in Llamas

**FIGURE 4:**
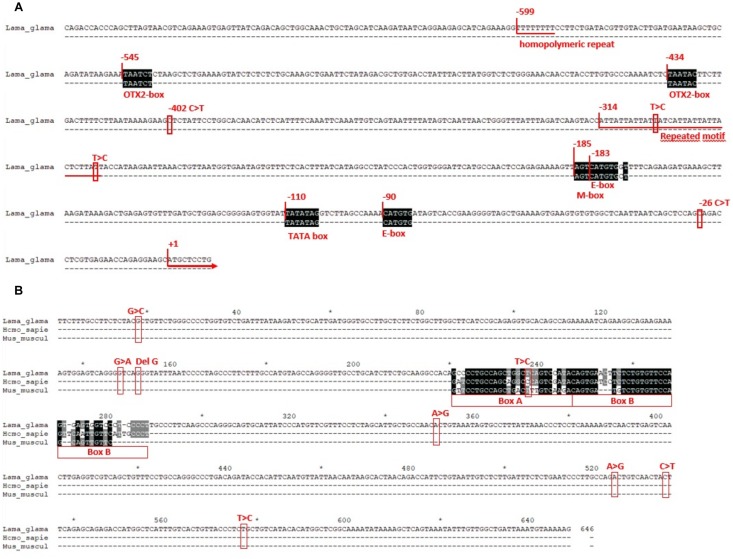
**(A)** Proximal promoter region of *TYR* gene. Positions +1 were assigned to the translation initiation site (ATG codon). Regulatory elements are aligned with its consensus sequence in black blocks and named underneath. Numbers above the elements indicate relative positions. Red squares indicate the location of the SNPs found in llamas and the repeated elements are underlined. **(B)** TYR LCR region. Nucleotide positions (from 1 to 646) are indicated in black above the sequence. Number 1 was arbitrarily designed to the first nucleotide showed. Boxes A and B are aligned to the ones of mouse and human (X76647 and AY180962) and the variation found in llamas is squared in red color.

*TYR* proximal promoter (GenBank accession number MK847855) showed three conserved elements, two E-boxes (CATGTG) and one M-box (AGTCATGTGCT), which are known binding sites for bHLH-LZ transcription factors, like MITF. It also presented two binding sites for the orthodenticle homeobox 2 (OTX2), which is another transcription factor that is not involved in melanogenesis, but it is important in the retinal pigment epithelium. Additionally, a sequence corresponding to the TATA box was identified between 110 and 104 bp upstream of the first codon. No variation was observed within these regulatory elements (Figure 4A).

Besides the SNP c.1-26C > T, already described, another C to T SNP was detected at −402 bp, where the three possible genotypes were observed in the different phenotypic groups. Additionally, two other polymorphisms were detected in less frequency: an homopolymeric repeat of nine thymidine, that was found to be heterozygote for a T deletion in three llamas, one of each phenotypic group; and an imperfect repeated motif of ATT, that commonly presented 11 repetitions except for two individuals, one diluted and one non-diluted, that presented one repeat more in heterozygosity.

Figure 4B shows the LCR region (GenBank accession number MK847856). The position of Boxes A and B, corresponding to the putative binding sites for transcription factors, are indicated and aligned to the ones of mouse and human (X76647 and AY180962). Polymorphisms found in this region, SNPs and a 1 bp deletion, are also marked in Figure 4B. All of them were located outside Boxes A and B except for a T to C transition, within Box A. However, that position is not conserved between human and mouse, since one presents a C and the other a T, as it was observed in llamas. As for the other polymorphisms, different genotypes were observed among the different color phenotypes.

## Discussion

The structure of the llama *TYR* gene was similar to that previously described in human and other species (García-Borrón and Solano, 2002; Kanteev et al., 2015); the exons were relatively conserved and so were the domains of the encoded protein. Furthermore, identity values compared to orthologous proteins were high.

In reference to genetic variation, llamas presented several polymorphisms along *TYR* gene that included different types of variation. Nevertheless, exons 3 and 4, which codify the active region of the enzyme, did not present any mutations.

Different repeated sequences were observed in non-coding regions of *TYR* gene: microsatellites and homopolymeric repeats. Although it is not completely understood yet, there is strong evidence that repeated elements do have a role in the genome and that their distribution is not random (Vieira et al., 2016). There are numerous studies indicating that repeats located in introns can affect gene transcription, mRNA splicing, or export to cytoplasm (Li et al., 2004). Moreover, some of these repeats can have an effect on the phenotype and the length of microsatellites is particularly important in this aspect (Sjakste et al., 2013). Long alleles are associated to high predisposition to pathologies (Belguith-Maalej et al., 2013). In relation to coat color, a recent study revealed the presence of a large intronic insertion in Tyrosinase Associated Protein 1 (*TYRP1*) of the American Mink that alters the splicing of the gene and produces the American Palomino phenotype (Cirera et al., 2016). Due to the complexity of the repeated sequences observed in llamas, that variated in length and in motif, it was not possible to determine the total number of alleles. A more thorough study should be carried out to analyze these variants in particular and their relation with coat color. Nevertheless, we considered important to describe them as detailed as possible since there are few microsatellites described for camelids in comparison with other species and they have important applications for other studies such as linkage analysis, evolution, forensics, and population genetics.

Usually, disrupting mutations in *TYR* gene, like frameshifts or nonsense mutations, result in an inactive truncated protein and have been reported to produce albino animals in different species (Oetting, 2000; Schmutz et al., 2004; Blaszczyk et al., 2005; Imes et al., 2006; Anistoroaei et al., 2008). Hypopigmentation, on the other side, has been more frequently associated with missense point mutations. Accordingly, there are at least seven alleles described that result in hypopigmentation in mice (Challa et al., 2016). Minks also present the Himalayan allele, that produces an hypopigmented phenotype and is the result of a single-nucleotide substitution (Benkel et al., 2009). So are the mutations associated with the diluted phenotypes Siamese and Burmese in cats (Lyons et al., 2005). We have analyzed TYR gene in llamas with non-diluted, diluted, and white phenotypes and three non-synonymous SNPs were observed. Although *in silico* analysis predicted the three SNPs to produce benign changes in the protein, the frequency of the G allele from c.428A > G was significantly higher in white compared to non-diluted, suggesting phenotype association. This allele produces an amino acid change from histidine to arginine in residue 143 of tyrosinase. Even though it is a relatively conserved position among species, the homologous protein from vampire bats (*D. rotundus*) presents an arginine in this position. Additionally, we searched the human data base from The Ensembl project^6^ and found that the same mutation was observed with a very low frequency (0.00006153), but it was not reported if there is an associated phenotype. Tanking into account all the information just mentioned, it is difficult to assess the possible functional relevance of this variant.

Besides the study of polymorphisms, we focused on the expression of *TYR* gene. We found that *TYR* was significantly less expressed in the WHITE group compared to NON-DILUTED. This was expected, since *MITF-M* gene, which regulates *TYR* expression, was found to be less expressed in white llamas compared to colored ones (Anello et al., 2019). Our results are also consistent with the observations of Paterson et al. (2015), who demonstrated that partial depletion of *TYR* inhibited melanosome maturation and the expression of genes that regulates melanogenesis, altering mouse coat color.

The expression level of *TYR* was also studied by Munyard (2011), who observed that *TYR*, together with seven other color-related genes, was expressed in a common pattern in alpacas: high in black, moderate in bay, and low in white. Similarly, Chen et al. (2012) analyzed the expression of *TYR* in the skin of Jining gray goats and it resulted higher in the dark-gray goats compared to the light-gray ones. Kim et al. (2014) also observed that *TYR* was more expressed in the dark vs. light muzzle of native Korean cows. In our study, the expression in the DILUTED group was in between the other two groups, although differences were not statistically significant. This could be due to a small difference in expression that would need a larger sample to be detectable. Additionally, differences in the degree of dilution of the animals that conformed this group might have influenced this result.

It is commonly known that UTR regions have important regulatory elements that control gene expression (Matoulkova et al., 2012). Therefore, the most important regulatory regions of *TYR* gene were analyzed in this study: the proximal promoter and the LCR. However, all the regulatory elements previously described within these regions were found to be conserved in llamas; the detected variation was located outside the elements. None of the polymorphisms observed in these regions seemed to explain the expression differences observed between white and colored animals.

Unexpectedly c.1-26C > T presented a significantly higher frequency of the C/T genotype in diluted animals compared to the other phenotypic groups. Nevertheless, this polymorphism alone is not causative for the color dilution, since C/T genotype was also observed in non-diluted llamas, where tyrosinase activity is expected to be normal. One possible explanation for this result could be that the same *TYR* mutation under different genetic backgrounds produces different phenotypes, as it was proposed for rabbits of different strains (Aigner et al., 2000). Additionally, different polymorphisms could be contributing together with c.1-26C > T to the final color phenotype. Finally, we cannot exclude that another polymorphism linked to c.1-26C > T, located in regions non-contemplated in this study (like intronic or other regulatory regions) is the actual causal of melanin dilution.

In this study, we have characterized the structure of *TYR* gene and its variation, contributing to the genetic knowledge of the llama. Moreover, we have analyzed the role of *TYR* variation and its expression in different color phenotypes, bringing new information to the understanding of the llama pigmentation mechanisms.

## Author Contributions

MA, LV-R, and FDR conceived and designed the research. MA and EF performed the experiments. MA, EF, and MD analyzed the data. MA and FDR wrote the manuscript. EF, MD, and LV-R revised the manuscript. All authors read and approved the final manuscript.

## Conflict of Interest Statement

The authors declare that the research was conducted in the absence of any commercial or financial relationships that could be construed as a potential conflict of interest.

## Footnotes

1 http://www.ifpcs.org/albinism/oca1mut.html

2 http://www.cbs.dtu.dk/services/TMHMM

3 http://genetics.bwh.harvard.edu/pph2/

4 https://sift.bii.a-star.edu.sg/

5 http://provean.jcvi.org/seq_submit.php

6 http://grch37.ensembl.org/Homo_sapiens/Gene/Variation_Gene/Table?db=core;g=ENSG00000077498;r=11:88910620-89028927
