# Supplementary figures and images for: TYR Gene in Llamas: Polymorphisms and Expression Study in Different Color Phenotypes
Source: Front Genet. 2019 Jun 12;10:568. doi: 10.3389/fgene.2019.00568 (PMC6582663; doi:10.3389/fgene.2019.00568)

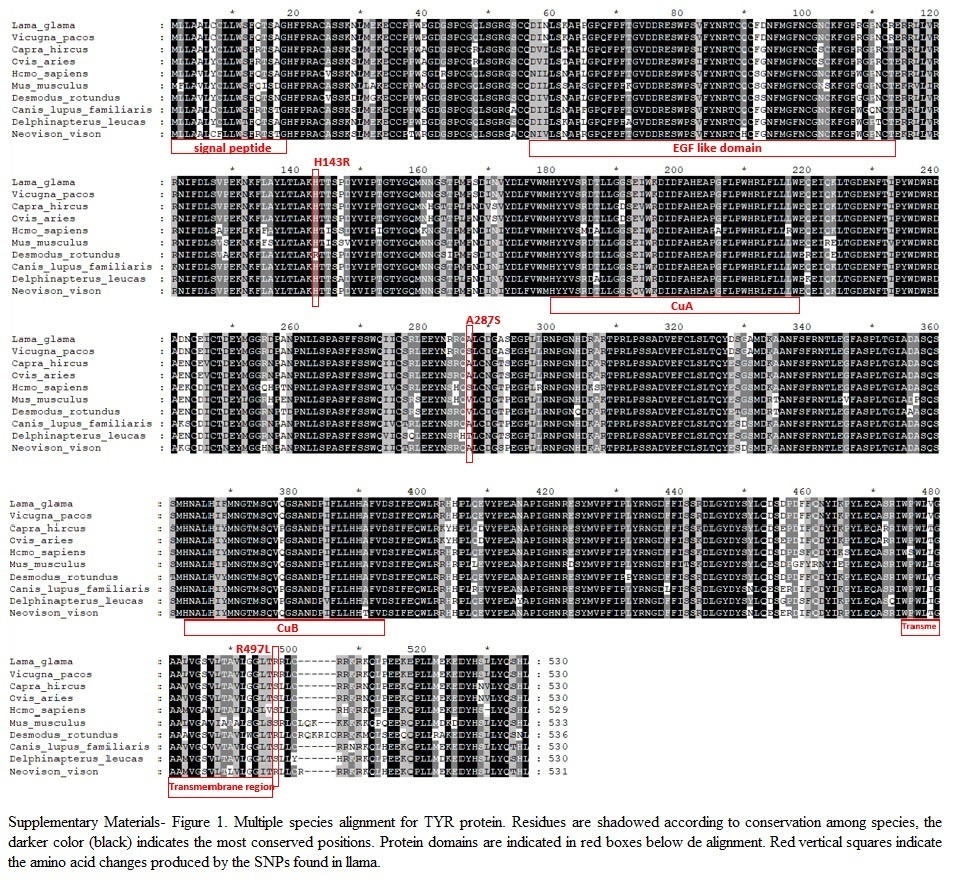

Supplement: Supplementary file 1 [file Image_1.JPEG]

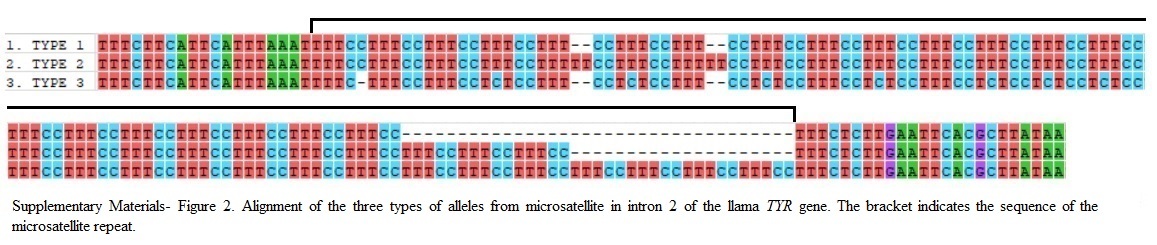

Supplement: Supplementary file 2 [file Image_2.JPEG]
