# Supplementary material for: TYR Gene in Llamas: Polymorphisms and Expression Study in Different Color Phenotypes
Source: Front Genet. 2019 Jun 12;10:568. doi: 10.3389/fgene.2019.00568 (PMC6582663; doi:10.3389/fgene.2019.00568)
Supplement: Supplementary file 3 [file Table_1.DOCX]

Supplementary Material

*TYR* gene in llamas: polymorphisms and expression study in different color phenotypes

**Melina Anello^1^, Estefanía Fernandez^1^, M. Silvana Daverio^1,2^, Lidia Vidal Rioja^1^ Florencia Di Rocco^1*^**

^1^Laboratorio de Genética Molecular, Instituto Multidisciplinario de Biología Celular (IMBICE), CONICET-UNLP-CIC, La Plata, Argentina.

^2^Cátedra de Biología, Departamento de Ciencias Biológicas, Facultad de Ciencias Exactas, Universidad Nacional de La Plata. La Plata, Argentina.

*** Correspondence:**Corresponding Author
fdirocco@imbice.gov.ar

Supplementary Materials- Table 1. Additional information about samples used in this study.

| Sample ID | Phenotypic group | Phenotypic subgroup | Origin |
| --- | --- | --- | --- |
| 33 | NON DILUTED | BLACK | Cusi Cusi, Jujuy |
| 51 | NON DILUTED | BLACK | Cusi Cusi, Jujuy |
| 76 | NON DILUTED | BLACK | Laguna Blanca, Catamarca |
| 78 | NON DILUTED | BLACK | Laguna Blanca, Catamarca |
| 86 | NON DILUTED | BLACK | Laguna Blanca, Catamarca |
| 87 | NON DILUTED | BLACK | Laguna Blanca, Catamarca |
| 95 | NON DILUTED | BLACK | Villaguay, Entre Ríos |
| 147 | NON DILUTED | BLACK | Abra Pampa, Jujuy |
| 151 | NON DILUTED | BLACK | Abra Pampa, Jujuy |
| 152 | NON DILUTED | BLACK | Abra Pampa, Jujuy |
| 159 | NON DILUTED | BLACK | Laguna Blanca, Catamarca |
| 167 | NON DILUTED | BLACK | Laguna Blanca, Catamarca |
| 194 | NON DILUTED | BLACK | La Plata, Buenos Aires |
| 195 | NON DILUTED | BLACK | La Plata, Buenos Aires |
| 200 | NON DILUTED | BLACK | Ciudad de Buenos Aires, Buenos Aires |
| 212 | NON DILUTED | BLACK | Ciudad de Buenos Aires, Buenos Aires |
| 215 | NON DILUTED | BLACK | Ciudad de Buenos Aires, Buenos Aires |
| 229 | NON DILUTED | BLACK | Abra Pampa, Jujuy |
| 287 | NON DILUTED | BLACK | La Carolina, San Luis |
| 301 | NON DILUTED | BLACK | La Carolina, San Luis |
| 339 | NON DILUTED | BLACK | La Carolina, San Luis |
| 346 | NON DILUTED | BLACK | La Carolina, San Luis |
| 356 | NON DILUTED | BLACK | La Carolina, San Luis |
| 42 | NON DILUTED | RED | Cusi Cusi, Jujuy |
| 82 | NON DILUTED | RED | Laguna Blanca, Catamarca |
| 83 | NON DILUTED | RED | Laguna Blanca, Catamarca |
| 84 | NON DILUTED | RED | Laguna Blanca, Catamarca |
| 85 | NON DILUTED | RED | Laguna Blanca, Catamarca |
| 99 | NON DILUTED | RED | Villaguay, Entre Ríos |
| 160 | NON DILUTED | RED | Laguna Blanca, Catamarca |
| 197 | NON DILUTED | RED | La Plata, Buenos Aires |
| 202 | NON DILUTED | RED | Ciudad de Buenos Aires, Buenos Aires |
| 211 | NON DILUTED | RED | Ciudad de Buenos Aires, Buenos Aires |
| 73 | NON DILUTED | BLACK FACE | Laguna Blanca, Catamarca |
| 100 | NON DILUTED | BLACK FACE | Villaguay, Entre Ríos |
| 139 | NON DILUTED | BLACK FACE | Abra Pampa, Jujuy |
| 142 | NON DILUTED | BLACK FACE | Abra Pampa, Jujuy |
| 143 | NON DILUTED | BLACK FACE | Abra Pampa, Jujuy |
| 153 | NON DILUTED | BLACK FACE | Abra Pampa, Jujuy |
| 158 | NON DILUTED | BLACK FACE | Laguna Blanca, Catamarca |
| 161 | NON DILUTED | BLACK FACE | Laguna Blanca, Catamarca |
| 163 | NON DILUTED | BLACK FACE | Laguna Blanca, Catamarca |
| 164 | NON DILUTED | BLACK FACE | Laguna Blanca, Catamarca |
| 168 | NON DILUTED | BLACK FACE | Laguna Blanca, Catamarca |
| 217 | NON DILUTED | BLACK FACE | Abra Pampa, Jujuy |
| 334 | NON DILUTED | BLACK FACE | La Carolina, San Luis |
| 7 | DILUTED | FAWN | Laguna de Pozuelos, Jujuy |
| 106 | DILUTED | FAWN | Villaguay, Entre Ríos |
| 146 | DILUTED | FAWN | Abra Pampa, Jujuy |
| 154 | DILUTED | FAWN | Abra Pampa, Jujuy |
| 155 | DILUTED | FAWN | Abra Pampa, Jujuy |
| 187 | DILUTED | FAWN | La Plata, Buenos Aires |
| 189 | DILUTED | FAWN | La Plata, Buenos Aires |
| 193 | DILUTED | FAWN | La Plata, Buenos Aires |
| 199 | DILUTED | FAWN | Ciudad de Buenos Aires, Buenos Aires |
| 203 | DILUTED | FAWN | Ciudad de Buenos Aires, Buenos Aires |
| 205 | DILUTED | FAWN | Ciudad de Buenos Aires, Buenos Aires |
| 208 | DILUTED | FAWN | Ciudad de Buenos Aires, Buenos Aires |
| 213 | DILUTED | FAWN | Ciudad de Buenos Aires, Buenos Aires |
| 214 | DILUTED | FAWN | Ciudad de Buenos Aires, Buenos Aires |
| 258 | DILUTED | FAWN | Cafayate, Salta |
| 354 | DILUTED | FAWN | La Carolina, San Luis |
| 374 | DILUTED | FAWN | Ciudad de Buenos Aires, Buenos Aires |
| 425 | DILUTED | FAWN | Antofagasta de la Sierra, Catamarca |
| 434 | DILUTED | FAWN | Antofagasta de la Sierra, Catamarca |
| 435 | DILUTED | FAWN | Antofagasta de la Sierra, Catamarca |
| 144 | DILUTED | GREY | Abra Pampa, Jujuy |
| 145 | DILUTED | GREY | Abra Pampa, Jujuy |
| 157 | DILUTED | GREY | Abra Pampa, Jujuy |
| 231 | DILUTED | GREY | Abra Pampa, Jujuy |
| 232 | DILUTED | GREY | Abra Pampa, Jujuy |
| 387 | DILUTED | GREY | Abra Pampa, Jujuy |
| 419 | DILUTED | GREY | Antofagasta de la Sierra, Catamarca |
| 8 | WHITE | BLUE-EYED-WITHE | Laguna de Pozuelos, Jujuy |
| 9 | WHITE | BLUE-EYED-WITHE | Laguna de Pozuelos, Jujuy |
| 10 | WHITE | BLUE-EYED-WITHE | Laguna de Pozuelos, Jujuy |
| 15 | WHITE | WHITE | Laguna de Pozuelos, Jujuy |
| 21 | WHITE | WHITE | Laguna de Pozuelos, Jujuy |
| 31 | WHITE | WHITE | Cusi Cusi, Jujuy |
| 35 | WHITE | WHITE | Cusi Cusi, Jujuy |
| 50 | WHITE | WHITE | Cusi Cusi, Jujuy |
| 79 | WHITE | WHITE | Laguna Blanca, Catamarca |
| 88 | WHITE | WHITE | Villaguay, Entre Ríos |
| 89 | WHITE | WHITE | Villaguay, Entre Ríos |
| 133 | WHITE | WHITE | Abra Pampa, Jujuy |
| 135 | WHITE | WHITE | Abra Pampa, Jujuy |
| 137 | WHITE | WHITE | Abra Pampa, Jujuy |
| 149 | WHITE | WHITE | Abra Pampa, Jujuy |
| 150 | WHITE | WHITE | Abra Pampa, Jujuy |
| 169 | WHITE | WHITE | Antofagasta de la Sierra, Catamarca |
| 170 | WHITE | WHITE | Antofagasta de la Sierra, Catamarca |
| 172 | WHITE | WHITE | Antofagasta de la Sierra, Catamarca |
| 174 | WHITE | WHITE | Antofagasta de la Sierra, Catamarca |
| 177 | WHITE | WHITE | Antofagasta de la Sierra, Catamarca |
| 178 | WHITE | WHITE | Antofagasta de la Sierra, Catamarca |
| 179 | WHITE | WHITE | Antofagasta de la Sierra, Catamarca |
| 180 | WHITE | WHITE | Antofagasta de la Sierra, Catamarca |
| 181 | WHITE | WHITE | Antofagasta de la Sierra, Catamarca |
| 182 | WHITE | WHITE | Antofagasta de la Sierra, Catamarca |
| 183 | WHITE | WHITE | Antofagasta de la Sierra, Catamarca |
| 184 | WHITE | WHITE | Antofagasta de la Sierra, Catamarca |
| 185 | WHITE | WHITE | Antofagasta de la Sierra, Catamarca |
| 196 | WHITE | WHITE | La Plata, Buenos Aires |
| 198 | WHITE | WHITE | Ciudad de Buenos Aires, Buenos Aires |
| 201 | WHITE | WHITE | Ciudad de Buenos Aires, Buenos Aires |
| 204 | WHITE | WHITE | Ciudad de Buenos Aires, Buenos Aires |
| 241 | WHITE | WHITE | Abra Pampa, Jujuy |
| 242 | WHITE | WHITE | Abra Pampa, Jujuy |
| 243 | WHITE | WHITE | Abra Pampa, Jujuy |
| 244 | WHITE | WHITE | Abra Pampa, Jujuy |
| 326 | WHITE | WHITE | La Carolina, San Luis |
| 338 | WHITE | WHITE | La Carolina, San Luis |
| 381 | WHITE | WHITE | Marcos Paz, Buenos Aires |
