# Supplementary material for: TYR Gene in Llamas: Polymorphisms and Expression Study in Different Color Phenotypes
Source: Front Genet. 2019 Jun 12;10:568. doi: 10.3389/fgene.2019.00568 (PMC6582663; doi:10.3389/fgene.2019.00568)
Supplement: Supplementary file 4 [file Table_2.DOCX]

Supplementary Material

*TYR* gene in llamas: polymorphisms and expression study in different color phenotypes

**Melina Anello^1^, Estefanía Fernandez^1^, M. Silvana Daverio^1,2^, Lidia Vidal Rioja^1^ Florencia Di Rocco^1*^**

^1^Laboratorio de Genética Molecular, Instituto Multidisciplinario de Biología Celular (IMBICE), CONICET-UNLP-CIC, La Plata, Argentina.

^2^Cátedra de Biología, Departamento de Ciencias Biológicas, Facultad de Ciencias Exactas, Universidad Nacional de La Plata. La Plata, Argentina.

*** Correspondence:**Corresponding Author
fdirocco@imbice.gov.ar

Supplementary Materials-Table 2. List of primers

| Primer name | Primer sequence (5'-3') | Amplification target | Amplicon lenght (bp) | T annealing (°C) |
| --- | --- | --- | --- | --- |
| TYREX1-F | CCGAAGGGGTAGCTGAAAAGT | TYR exon 1 | 958 | 60 |
| TYREX1-R | GCCAGAAGAGGCGATTGCTA |  |  |  |
| TYREX2-F | ACCTGGAGGAGGAGACAGCA | TYR exon 2 | 521 | 65 |
| TYREX2-R | ACCCCGCTAGGGTTATTGGC |  |  |  |
| TYREX3-F | GGCACCCAGAATGTGAAGAA | TYR exon 3 | 363 | 58 |
| TYREX3-R | ACTGCATTTAAAACCAACAAACA |  |  |  |
| TYREX4-F | TTGGCATCTGTCCAGGGCTT | TYR exon 4 | 541 | 60 |
| TYREX4-R | AGTGCTCTTCCCACAGTTGC |  |  |  |
| TYREX5-F | GCGCCTGACTCCAAAGTGC | TYR exon 5 | 441 | 62 |
| TYREX5-R | AGGCCAAGGATGTCTGCTGG |  |  |  |
| TYR-26C-F | TGTGGCTCAATTAATCAGCTCAAGC | c.1-26C>T allele C | 140 | 63 |
| TYR-26C-R | CAGGTTCTTGGAGGAGGCACA |  |  |  |
| TYR-26T-F | TGTGGCTCAATTAATCAGCTCAAGT | c.1-26C>T allele T | 183 | 63 |
| TYR-26T-R | GACTCCCATCACCCTCCCAC |  |  |  |
| TYR1490G-F | GTGCTGGGTGGGCTCAATCG | c.1490G>T allele G | 164 | 60 |
| TYR1490G-R | CGAGGCCAGGCTTTTTGACCC |  |  |  |
| TYR1490T-F | GTGCTGGGTGGGCTCAATCT | c.1490G>T allele T | 105 | 60 |
| TYR1490T-R | CAGCAGGCTGTGGTAATCCTCC |  |  |  |
| TYR-PPROX-F | CCCCAAATAAAGCAGACCACCC | TYR proximal promoter | 792 | 65 |
| TYR-PPORX-R | TTCTTGGAGGAGGCACAGGC |  |  |  |
| TYR-LCR-F | TTGTGAAAAACACAGACTAGGCA | TYR-LCR | 789 | 58 |
| TYR-LCR-R | TTACCCAGTGTGAAGCCTCG |  |  |  |
| qTYR-F | CCTGAAGCCAATGCACCCAT | qPCR- TYR mRNA | 140 | 60 |
| qTYR-R | TCCGGGTCTGAATCTTGTAGGT |  |  |  |
| 18S F | GTAACCCGTTGAACCCCATT | qPCR- 18S mRNA | 150 | 60 |
| 18S R | CCATCCAATCGGTAGTAGCG |  |  |  |
