# Supplementary material for: TYR Gene in Llamas: Polymorphisms and Expression Study in Different Color Phenotypes
Source: Front Genet. 2019 Jun 12;10:568. doi: 10.3389/fgene.2019.00568 (PMC6582663; doi:10.3389/fgene.2019.00568)
Supplement: Supplementary file 5 [file Table_3.DOCX]

Supplementary Material

*TYR* gene in llamas: polymorphisms and expression study in different color phenotypes

**Melina Anello^1^, Estefanía Fernandez^1^, M. Silvana Daverio^1,2^, Lidia Vidal Rioja^1^ Florencia Di Rocco^1*^**

^1^Laboratorio de Genética Molecular, Instituto Multidisciplinario de Biología Celular (IMBICE), CONICET-UNLP-CIC, La Plata, Argentina.

^2^Cátedra de Biología, Departamento de Ciencias Biológicas, Facultad de Ciencias Exactas, Universidad Nacional de La Plata. La Plata, Argentina.

*** Correspondence:**Corresponding Author
fdirocco@imbice.gov.ar

Supplementary Materials-Table 3. Alleles observed in homozygosity for intron 2 microsatellite.

|  | allele motif | n° repeats |
| --- | --- | --- |
| type 1 | (TTTCC)20 | 20 |
|  | (TTTCC)21 | 21 |
|  | (TTTCC)23 | 23 |
| type 2 | (TTTCC)12-(TT)-(TTTCC)2-(TT)-(TTTCC)14 | 28 |
|  | (TTTCC)9-(TT)-(TTTCC)2-(TT)-(TTTCC)16 | 27 |
|  | (TTTCC)6-(TT)-(TTTCC)2-(TT)-(TTTCC)22 | 30 |
|  | (TTTCC)6-(TT)-(TTTCC)2-(TT)-(TTTCC)14 | 22 |
|  | (TTTCC)4-(TT)-(TTTCC)2-(TT)-(TTTCC)18 | 24 |
| type 3 | (TTTC)-(TTTCC)2-[(TCTCC)-(TTTCC)]4- (TCTCC)3-(TTTCC)14 | 28 |
