# Supplementary material for: TYR Gene in Llamas: Polymorphisms and Expression Study in Different Color Phenotypes
Source: Front Genet. 2019 Jun 12;10:568. doi: 10.3389/fgene.2019.00568 (PMC6582663; doi:10.3389/fgene.2019.00568)
Supplement: Supplementary file 6 [file Table_4.DOCX]

Supplementary Material

*TYR* gene in llamas: polymorphisms and expression study in different color phenotypes

**Melina Anello^1^, Estefanía Fernandez^1^, M. Silvana Daverio^1,2^, Lidia Vidal Rioja^1^ Florencia Di Rocco^1*^**

^1^Laboratorio de Genética Molecular, Instituto Multidisciplinario de Biología Celular (IMBICE), CONICET-UNLP-CIC, La Plata, Argentina.

^2^Cátedra de Biología, Departamento de Ciencias Biológicas, Facultad de Ciencias Exactas, Universidad Nacional de La Plata. La Plata, Argentina.

*** Correspondence:**Corresponding Author
fdirocco@imbice.gov.ar

Supplementary Materials-Table 4. Alleles and genotypes observed for intron 4 microsatellite.

| alleles observed in homozygosity | n° repeats |
| --- | --- |
| (GAT)13 | 13 |
| (GAT)5-(AAT)-(GAT)7 | 13 |
| (GAT)5-(AAT)-(GAT)9 | 15 |
| genotypes observed | possible combination of alleles |
| (GAT)5-(RAT)-(GAT)7 | (GAT)13 + (GAT)5-(AAT)-(GAT)7 |
| (GAT)4-(RAT)2-(GAT)7 | (GAT)13 + unknown allele |
| (GAT)3-(RAT)-(GAT)-(RAT)-(GAT)7 | (GAT)5-(AAT)-(GAT)7 + (GAT)5-(AAT)-(GAT)9 |
