# Supplementary material for: TYR Gene in Llamas: Polymorphisms and Expression Study in Different Color Phenotypes
Source: Front Genet. 2019 Jun 12;10:568. doi: 10.3389/fgene.2019.00568 (PMC6582663; doi:10.3389/fgene.2019.00568)
Supplement: Supplementary file 7 [file Table_5.DOCX]

Supplementary Material

*TYR* gene in llamas: polymorphisms and expression study in different color phenotypes

**Melina Anello^1^, Estefanía Fernandez^1^, M. Silvana Daverio^1,2^, Lidia Vidal Rioja^1^ Florencia Di Rocco^1*^**

^1^Laboratorio de Genética Molecular, Instituto Multidisciplinario de Biología Celular (IMBICE), CONICET-UNLP-CIC, La Plata, Argentina.

^2^Cátedra de Biología, Departamento de Ciencias Biológicas, Facultad de Ciencias Exactas, Universidad Nacional de La Plata. La Plata, Argentina.

*** Correspondence:**Corresponding Author
fdirocco@imbice.gov.ar

Supplementary Materials-Table 5. Distribution of haplotypes among phenotypic groups

|  | AGG/AGG | AGT/AGT | ATG/ATG | AGG/AGT | AGG/GGG | AGG/ATG | ATG/ATT | GTG/GTG | GGG/GGG | ATT/AGT |
| --- | --- | --- | --- | --- | --- | --- | --- | --- | --- | --- |
|  |  |  |  |  |  |  |  |  |  |  |
| NON-DILUTED PHENOTYPES | 19 | 2 | 5 | 10 | 2 | 4 | 2 | - | - | 1 |
| DILUTED PHENOTYPES | 7 | 2 | 3 | 8 | 1 | - | - | - | - | - |
| WHITE PHENOTYPES | 12 | 1 | 4 | 9 | 5 | 3 | 1 | 1 | 1 | - |
| TOTAL | 38 | 5 | 12 | 27 | 8 | 7 | 3 | 1 | 1 | 1 |
